# Employers’ perceptions of breastfeeding practice of employed mothers in Addis Ababa, Ethiopia: a qualitative study

**DOI:** 10.1186/s13006-022-00482-9

**Published:** 2022-05-23

**Authors:** Firmaye Bogale Wolde, Jemal Haidar, Yalemwork Getnet

**Affiliations:** 1 Ethiopian Public Health Institute, Addis Ababa, Ethiopia; 2 School of Public Health, Addis Ababa University, Addis Ababa, Ethiopia

**Keywords:** Employer, Breastfeeding, Work environment, Mother-friendly, Employed mothers

## Abstract

**Background:**

The Ethiopian Demographic and Health Survey 2019 shows that 59% of children are exclusively breastfed for the first 6 months of life, then the rate decreases sharply with age. Nearly half of the Ethiopian labor force (46%) is comprised of women. This is encouraging since women’s employment is one way of ensuring women’s empowerment. However, various factors related to employment make it one of the commonly mentioned factors contributing for the low prevalence of breastfeeding. Hence, there needs to be a conducive work environment that accommodates maternal needs to not fall back from empowerment and to improve breastfeeding practice. There are not many studies in Ethiopia that focus on work environment in relation with employers’ experience and their perception of breastfeeding of employed mothers. Therefore, this study aims to explore employers’ experience and perception of employed mothers’ breastfeeding experience in different working environments in Addis Ababa, Ethiopia.

**Methods:**

A qualitative study design using a descriptive phenomenology strategy was employed in this study and purposive sampling technique was used to recruit study participants. Data was collected between December 2016 and May 2017 in Addis Ababa city from 10 employers from private, governmental and non-governmental institutions through an in-depth interview. Thematic data analysis was performed where collected data was organized, coded and categorized into themes to give meaningful contributions to answering the research questions.

**Results:**

Understanding breastfeeding, current maternity leave, perception of breastfeeding supporting the conditions and mother-friendly work environment were the themes generated after analysis. Almost all employers in this study recognized the importance of breastfeeding despite their different work environments and they also acknowledged the importance of making the working environment mother-friendly for stability and motivation of employed mothers.

**Conclusions:**

Providing mothers with a friendly environment is understood as a positive thing by employers. The current maternity leave of 3 months has low acceptance and both onsite childcare center and six-month maternity leave are believed to help in creating a mother-friendly work environment despite their pros and cons.

## Background

The 2019 Ethiopian Demographic and Health Survey shows that 59% of children are exclusively breastfed for the first 6 months of life. The percentage of children who are exclusively breastfed sharply decreases with age from 73% of infants aged 0-1 month to 68% of children 2-3 months to 40% of infants aged 4-5 months. Additionally about 6% of children under the age of 6 months are not breastfed at all [1].

In Ethiopia, the involvement of women in the labor force has increased continuously in the past years and as of 2019 the World Bank report showed that 46.5% of the labor force is comprised of women [2]. Though women employment serves as a way of ensuring women’s empowerment, studies both at the global and Ethiopian settings show that maternal employment is one of the commonly mentioned factors that contribute to the discontinuation of breastfeeding [3–6]. With regards to employment of mothers, factors like employers’ knowledge and attitude towards breastfeeding, and support provided at the workplace are listed as some aspects that affect breastfeeding [6, 7]. Subsequently, studies in Ethiopia show breastfeeding prevalence of employed mothers is lower than unemployed mothers [8, 9].

It is essential to ensure the presence of a conducive work environment that supports employed mothers breastfeeding at work not to fall back from empowering women. Breastfeeding must be ensured for all mothers by viewing breastfeeding practice as a result of the combined effort of government, work environment, family and community, rather than putting it as the sole responsibility of the mother herself [10].

There are different studies on the breastfeeding experience of employed mothers in Ethiopia [11, 12]. There are also studies from other countries, that focus on exploring employers’ perception and experience of employed mothers’ breastfeeding in different work environments [13]. However, we were only able to find one study conducted in the Ethiopian setting [14]. This study, which is conducted by interviewing 15 managers, indicated the presence of positive attitude towards the importance of breastfeeding among managers. It also shows the concern of managers in relation to the effect of breastfeeding on staffing causing shortage of staff and concern on the performance of breastfeeding mothers. In addition, it is indicated that managers feel that the government should take the main responsibility in relation to breastfeeding policies and physical resources for breastfeeding at work places. This study only included participants from similar positions, which could have influenced the findings and it did not highlight the perspective of participants on the different options that could be presented to support breastfeeding mothers at working place [14].

Thus, exploring the general perceptions towards breastfeeding and specific outlooks of the possible options issue will not only show employers’ experience but will be a standing point to understand employers’ view and find suitable conditions for better breastfeeding practice.

Therefore, this study aims to explore employers’ experience and perception of employed mothers’ breastfeeding experience in different working environments in Addis Ababa, Ethiopia.

## Methods

### Design

A qualitative study design using a descriptive Phenomenology strategy was employed in this study, where studying a small number of subjects through extensive engagement to develop patterns and relationships of meaning is done [15].

### Participants

The study population in this study included employers and personnel from major stakeholders responsible for breastfeeding related issues like arrangement of maternity leave, work place policies of breastfeeding, and provisions to support breastfeeding women. This study included 10 employers from private, governmental and non-governmental institutions.

Purposive sampling technique was used to recruit study participants where human resource officers or directors of institutions in each organization helped to identify eligible participants in the organizations. Participants who were in an employer position, were responsible and had the authority to decide on issues regarding breastfeeding in the organizations under study were included.

### Data collection

Data collection was done through in-depth interview by using an interview guide that contained semi-structured and a few open-ended questions to have a high degree of flexibility.

Each interview question had a single core question that permitted participants to express what they felt while maintaining a central frame of inquiry. The interview had questions on general knowledge of employers on breastfeeding, the experience of employers on working with breastfeeding mothers, knowledge and perception on mother-friendly work environment and experience with regards to supporting conditions for breastfeeding in the workplace.

The interview guide was improved by arranging question orders, clarifying questions and adding missing probes by conducting pilot interviews. The pilots were conducted with interviewees who were potential participants working in governmental and private organizations as supervisors of breastfeeding mothers. Some missing probes were also added after the first two interviews which were found to bring in more information. All interviews were conducted by the principal investigator (FB) who worked in a governmental organization but did not have experience of acting as an employer of breastfeeding mothers.

The date, time, and place of each interview were arranged following what was most favorable and comfortable for the participants. All the interviews were audiotaped with the participant’s consent and the interviews took an average of an hour. Informed consent was taken before each interview where participants were informed about the purpose, procedures, benefits, risks and the right of the respondents to refuse to respond for a few or all of the questions.

Data were collected from employers who met the inclusion criteria from December 2016 to May 2017 in Addis Ababa the capital city of Ethiopia. Data were collected until information saturation was attained.

### Data analysis

Thematic data analysis was performed to describe and compare general statements as relationships and themes present on the data according to the method of analysis described by Kleiman [16].

The interviews of this study were digitally recorded, transcribed verbatim and translated. The first stage of data analysis involved reading and re-reading each transcript and listening to the audio recording.

Initial coding was done in the second stage by reading the transcript in-depth and making note of anything important and paying attention to where and how patterns occurred. Thirdly, codes were categorized into themes followed by searching for connections and meaning of emergent themes. The final stage involved generating clear definitions of each theme and their meaningful contributions to answering the research question.

During data collection, respondent validation was done by restating and paraphrasing information for respondents to determine the accuracy, and participants were also revisited for transcript review before data analysis which gave better clarity on the data. Reliability was ensured by trying to avoid leading questions and all transcripts were cross-checked with the oral discourse for consistency, and transcripts and codes were constantly compared with the data to prevent drift of definition of codes [17]. The analysis was done manually and rigor was enhanced through regular discussions between authors who read all interview transcripts, counter checked the transcripts, coded the data and agreed on the emerging themes by going through the data.

### Operational definition

The term employer in this study refers to a person responsible, directly or indirectly, for decisions on breastfeeding issues of an employed breastfeeding mother and supporting conditions provided.

Supporting condition at work indicates to a working condition that has provisions to enable employees to meet both their family and work commitments. Accordingly, supporting condition at work refers to working environment that either has onsite childcare center or provides 6 months of maternity leave to mothers.

## Results

### Employers’ demographic characteristics

Three male and seven female employers who came from a range of positions that made them responsible for issues of employed mothers, breastfeeding and supporting conditions in the work place were involved in this study.

Three of the employers were from organizations that offer onsite childcare center (EO), two employers came from an organization that offer six-month maternity leave (ES) and five employers were from organizations with none of the two supporting conditions (EN) (Table 1).

**Table 1:** Employers’ basic characteristics, Addis Ababa, Ethiopia, 2017

No	Sex	Occupation	Organization
1	F	Human resource coordinator	Health Institution, Government
2	M	Health department head	Education Institute. Government
3	M	Human resource coordinator	Insurance Company, Private
4	F	Women’s affair officer	State Enterprise, Government
5	F	Daycare coordinator	Technology Group, Private
6	F	Manpower corporate director	Technology Group, Private
7	M	Health and nutrition department coordinator	International NGO, Private
8	F	Child support & inspection director	Bureau of Women’s And Youth Affair, Government
9	F	Women’s affair bureau director	Bureau of Women’s And Youth Affair, Government
10	F	MCH and nutrition department coordinator	Federal Ministry of Health, Government

Four themes were generated from the analysis of the data from interviews

i) Understanding breastfeeding.
ii) Current maternity leave.
iii) Perception of breastfeeding supporting conditions.
iv) Mother-friendly work environment

### Understanding breastfeeding

Almost all employers except one expressed that they believe 6 months exclusive breastfeeding is needed and should also be continued to 2 years. One employer, however, explained that with the current living situation and employment it is enough if mothers breastfeed up to the age of one.

*“If you are asking me what I think, I say it’s enough for an employed mother to breastfeed her baby only in the morning and night time to about a year; given the current living situation.” En.*

Employers described the benefit of breastfeeding majorly related to health, psychosocial wellbeing*,* and economic benefit.

*“Breastfeeding has health benefits like a baby gets immunity from the first breast milk and breastfeeding doesn’t need out of pocket-expense. Plus it improves psychosocial health by increasing the bond between mother and child.” Es.*

They also expressed the impact of not breastfeeding on the development of children and the satisfaction of mothers.

*“In my opinion, if a mother doesn’t breastfeed she will primarily lose mental satisfaction and she won’t be able to care for her child which will hurt her child’s growth.” Es.*

### Current maternity leave

Regarding the acceptance of the current national law of maternity leave, which is 3 months, employers expressed that it is not accepted by employees because it doesn’t consider the actual living situation and it hurts mothers’ readiness for work. Additionally, it is explained that very few are in favor of the law because it contradicts with the recommendation of 6 months exclusive breastfeeding.

*“Readiness to work is lesser among the workers who return from maternity leave. I’m afraid this is because it is not balanced with the workload they have which hurts the mothers*.” *En.*

Employers also said mothers’ restlessness, tiredness and being stressed are because of the shortness of the maternity leave given. They also explained that mothers come back because of the lack of options and that this affects the work.

*“Even if a mother comes back to work she’ll be restless and weak because she stays awake at night to breastfeed. Honestly speaking, mothers come back because they have no other option.” En.*

All participants explained that the three-month leave has two sections which are prenatal leave of 1 month and postnatal leave of 2 months duration but that there is a difference in the provision of the leave.

Most of the employers said that it cannot be amended in any way; even if a mother gave birth before taking any of her maternity leave, she will only be given the 2 months postnatal leave. However, two respondents explained otherwise saying that a mother can take the whole three-month maternity leave after birth if she wants to.

*“According to the current proclamation, maternity leave is given one month before and two months after birth. The law doesn’t state the whole three months to be given after birth because it’ll cause irregularities on work.” Eo.*

According to the employers, annual leave is a solution mostly used to extend maternity leave and taking unofficial leave or break with the agreement of boss is also common.

*“I try to help mothers with difficulties by arranging unofficial supports like coming in late to work and leaving early.” En.*

Another way that mothers try and extend their stay at home, as described by employers, is taking leave without payment. They explained that mothers ask for this kind of leave when they have difficult situations and this kind of leave is granted according to the specific case a mother has. Employers also explained that this type of leave is generally not encouraged to prevent it from becoming a trend.

*“Few mothers might not return to work after using both maternity and annual leave due to special cases like a child’s health problem. Accordingly, mothers are given ‘leave without payment’ for one month or so. But we don’t encourage this practice because if it becomes a trend it will grow in to a norm.” En.*

### Perception of breastfeeding supporting conditions

Onsite childcare center is discussed as one of the options to solve problems an employed woman faces as a mother. Participants discussed that presence of an onsite childcare center ensures women productivity, happiness at work and stability.

*“When onsite childcare center is accessible mothers can do their job properly, become stable and happy.” En.*

Participants explained the fact that mothers would not need to quit their jobs to raise their child if this center is accessible, which is what happens in most cases.

*“The presence of a day care center will ease so many problems and mothers will not stay home or quit their job.” En.*

There are some concerns presented by employers concerning an onsite childcare center which mainly are the difficulty it poses for an organization regarding space and related costs of hiring a baby sitter and fulfilling necessary facilities for the center. But despite these issues employers emphasized that the benefit outweighs the cost.

*“As a facility what is thought as the cost of having an onsite child care center includes the space it takes and fulfilling all the important things to make it safe and comfortable for children. These children need someone to care for them which is additional cost to be covered by the facility.” En.*

The other thing mentioned is the issue of convenience for women coming from far and the quality and consistency in the long run.

*“We should insure that this place for children and mothers is permanent. The most common challenges are lack of space to build this center and making the service continuous with quality.” Es.*

Participants also explained that increment of female employee number might put extra pressure to the organization but it still has more advantage.

*The increment of female employees might bring changes like the need for hiring more baby sitters and expanding space because there will probably be more children. But the benefit of the center is still great.” Eo.*

One of the challenges mentioned by employers in institution that has onsite childcare center is the difficulty of getting the mothers familiar with a programmed breastfeeding and care for children.

*“One problem we faced was getting the mothers to breastfeed in the set out schedule. Mothers come just after finishing their leave and they are used to breastfeeding whenever they want which makes it hard for them to follow a schedule right away.” Eo.*

The other supporting condition discussed is six-month maternity leave. Employers discussed the contribution of six-month maternity leave in a sense that it increases productivity, focus on job and better breastfeeding trend.

*“If a woman comes back to work after getting a six-month maternity leave she’ll take care of major problems that she might face beforehand and she’ll be stable at work.” Es.*

Employers also expressed that it is a great opportunity for mothers to rehabilitate, especially for those on positions where a great deal of labor input is needed.

*“I prefer the six-month leave because it helps mothers to recover because mothers in this factory work on machines all day. Imagine, these mothers had been pregnant for nine months and lost a lot of blood plus they breastfeed.” Eo.*

The things raised as a concern by employers include the need and the difficulty of getting a replacement employee for the 6 months and the impact it has on the operation of the work.

*“I think the issue here is the need for a person that fills in a mother’s position when she is not around. This could mean additional arrangement of hiring replacement personnel.” En.*

Another concern is related with difficulty of implementation because of lesser acceptance by private sectors and the probability of employers being inclined to hiring men over women.

*“Private sectors might not be willing to implement six-month maternity leave and even if it they accept it as an obligation there will be high tendency of getting women out of the business using subtle reasons.” Es.*

### Mother-friendly work environment

The employers understand the term mother-friendly environment as being an environment that accommodates mother’s needs as a woman and a mother by giving time to her child in addition to her work. And they also said it should include an understanding staff and situations that are fitting to motherhood.

*“The first thing is that the environment should give time to children in addition to the work. Secondly, I think there should be a well-rounded waiting place for children who are not old enough to get in to school.” En.*

Employers also described that the environment should be beneficial to a mother and should fulfill her basic needs where she faces no problems because of pregnancy, being a woman or a mother.

*“Mother-friendly work environment is facilitating every vital thing for women during pregnancy and breastfeeding, like building a friendly place for mothers to breastfeed at work and making it functional.” En.*

Participants explained that creating a friendly work environment for mothers means in one way or another helping everyone else around her.

*“A healthy mother can raise a healthy child and a healthy generation. That’s why we need to push through as this is an issue helpful for all of us.” En.*

Employers also emphasized on the fact that a friendly environment ensures mental satisfaction of women, makes them effective, stable and decreases absenteeism which in turn helps the organization.

*“Creating a comfortable environment for a mother means creating a productive and stable employee. Mothers encounter different problems because of lack of enough care for their children that can be prevented by building a system that supports women and in turn decreases absenteeism.” Eo.*

## Discussion

The experience and perception of employers on mother-friendly work environment and breastfeeding of employed mothers in different work settings was explored by this study.

Employers in this study understood the importance of breastfeeding and the provision of women-friendly work environments, with different views on the two supporting conditions, i.e., onsite childcare center and six-month maternity leave.

All employers in this study explained the importance of breastfeeding for both children and mothers and almost all of them explained that a child should be breastfed for an exclusive 6 months and continue for 2 years. This is similarly explained by employers in other studies who pointed out the importance of breastfeeding for mothers and children, and that mothers who breastfeed miss less work because of fewer episodes of child sickness [18, 19].

Employers in this study understand mother-friendly work environment as a place that accommodates the needs of a mother in a whole-rounded way and they agree on its importance which is also supported by another study in Ethiopia emphasizing the importance of putting breastfeeding-friendly work environment as a necessity comprising worksite daycare center [8]. However, despite the agreement, only a few employers have supporting conditions for breastfeeding employees.

It is seen in this study that employers have witnessed that mothers are stable and more productive when provided with a supporting condition at the workplace. This is similarly expressed in other studies that showed providing support at workplace improved job satisfaction, decreased turnover and reduced insurance costs [20–23].

According to this study, employers’ preference for the type of supporting condition in the workplace differs, based on their perception of advantage and disadvantage of the specific supporting condition in the workplace. This is consistent with the result of a systematic review which showed that all employers implement a breastfeeding support program that fits their company’s budget and resources [24].

Ethiopia officially offers a three-month maternity leave which is often related to concerns of insufficient duration and non-uniform implementation, as explained by employers in this study. The presence of this non-uniform implementation has brought unofficial and differential treatment of employed mothers, which could be contributing to the fact that most mothers do not get back to work just after 3 months, which is also described by a study that conducted focus group discussion on maternity leave in Ethiopia [25]. Mothers either use their annual leave to lengthen their stay at home, or they sometimes bring sick leave to stay with their babies. This type of unexpectedly longer leave could bring more pressure on the work than planned maternity leave.

Regarding six-month maternity leave, employers raised the risk of extra cost and burden on work if several mothers go out on leave at the same time and stressed the related challenge to hire replacement employees just for 6 months. This can be linked with the concern of employers from stakeholder bureaus on how challenging it can be to implement the six-month maternity leave due to possible lack of acceptance by private sectors. Similarly, a study in India stated the potential abandonment of tasks in the workplace as well as a potential loss of some form of cash benefit that will be incurred because of lengthened leave [26].

Another supporting condition seen in this study is the onsite childcare center. The concerns raised were the added cost of hiring babysitters, the need for extra space to build the center and ensuring the consistency of the service. These are also expressed as barriers in other studies where employers indicated that private places for nursing mothers could prove costly and raise budgetary issues [18, 26]. In spite of these concerns, the majority of the employers described that an onsite childcare center is advantageous, as it improved mothers’ stability and productivity thereby decreasing employee turnover. Additionally, preplanning for these issues could solve them ahead of time.

Generally, employers with neither of the two supporting conditions and from the organization with six-month maternity leave, were more inclined to having onsite childcare center in the workplace considering the anticipated negative impact of longer maternity leave on the operation of an organization.

## Conclusions

Employers understood that providing a friendly work environment for employed mothers as beneficial. Even though both onsite childcare center and six-months maternity leave have their advantages and disadvantages, both are believed to help in creating a mother-friendly work environment better than the current three-month maternity leave.

Additionally, finding a conducive supporting condition by considering different working environments is needed. Further large scale quantitative studies are essential to substantiate the evidence generated from this study and look into the cost analysis of the different supporting conditions. It should also be taken into consideration that this study did not find more than one organization that provides a six-month maternity leave.

## Data Availability

The datasets used in this study are available from the corresponding author on reasonable request.

## Abbreviations

EN: Employers from institution that has neither onsite childcare center nor six-month maternity leave
EO: Employers from institution that has onsite childcare center
ES: Employers from institution that gives six-month maternity leave
NGO: Non-governmental organization
UNICEF: United Nations Children’s Fund
WHO: World Health Organization
